# Effect and Mechanism Analysis of Pig *FUT8* Gene on Resistance to *Escherichia coli* F18 Infection

**DOI:** 10.3390/ijms232314713

**Published:** 2022-11-25

**Authors:** Lisi Wu, Yifu Wang, Shenglong Wu, Zhengchang Wu, Wenbin Bao

**Affiliations:** 1 Key Laboratory for Animal Genetics, Breeding, Reproduction and Molecular Design of Jiangsu Province, College of Animal Science and Technology, Yangzhou University, Yangzhou 225009, China; 2 Joint International Research Laboratory of Agriculture & Agri-Product Safety, Yangzhou University, Yangzhou 225009, China

**Keywords:** pig, *FUT8*, *E. coli* F18, SNP, transcription factor

## Abstract

Post-weaning diarrhea caused by enterotoxigenic *Escherichia coli* F18 (*E. coli* F18) causes significant economic losses for pig producers. Fucosyltransferase 8 (*FUT8*) is a glycosyltransferase that catalyzes core fucosylation; however, its role in mediating the resistance to *E. coli* F18 infection in pigs remains unknown. In this study, we systematically verified the relationship between *FUT8* expression and *E. coli* resistance. The results showed that *FUT8* was expressed in all detected tissues of Meishan piglets and that its expression was significantly increased in the duodenum and jejunum of *E. coli* F18-sensitive individuals when compared to *E. coli* F18-resistant individuals. *FUT8* expression increased after exposure to *E. coli* F18 (*p* < 0.05) and decreased significantly after LPS induction for 6 h (*p* < 0.01). Then, the IPEC-J2 stable cell line with *FUT8* interference was constructed, and *FUT8* knockdown decreased the adhesion of *E. coli* F18ac to IPEC-J2 cells (*p* < 0.05). Moreover, we performed a comparative transcriptome study of IPEC-J2 cells after *FUT8* knockdown via RNA-seq. In addition, further expression verification demonstrated the significant effect of *FUT8* on the glycosphingolipid biosynthesis and Toll-like signaling pathways. Moreover, the core promoter of *FUT8*, which was located at −1213 bp to −673 bp, was identified via luciferase assay. Interestingly, we found a 1 bp C base insertion mutation at the −774 bp region, which could clearly inhibit the transcriptional binding activity of *C/EBPα* to an *FUT8* promoter. Therefore, it is speculated that *FUT8* acts in a critical role in the process of *E. coli* infection; furthermore, the low expression of *FUT8* is conducive to the enhancement of *E. coli* resistance in piglets. Our findings revealed the mechanism of pig *FUT8* in regulating *E. coli* resistance, which provided a theoretical basis for the screening of *E. coli* resistance in Chinese local pig breeds.

## 1. Introduction

Post-weaning diarrhea (PWD) is one of the most common major diseases for piglets, which, in turn, causes huge economic losses to the pig industry [1,2,3]. *Escherichia coli* is the most common pathogen of PWD in the swine industry. In order to prevent the disease, there has been an overuse of antibacterial drugs and antibiotics, which has led to some instances of antibacterial and antibiotic resistance in certain pathogens [4]. *Escherichia coli* is specifically attached to receptors on the surface of epithelial cells of the small intestine. It can cause immune reactions and pathological changes in intestinal cells, as well as cause diarrhea. It produces enterotoxins mainly through bacterial adhesins, resulting in immune responses and pathological changes in intestinal cells and, also, causing diarrhea. Therefore, the key to investigating the resistance of pigs to *E. coli* lies in the expression of the F18 receptor, the integrity of the intestinal barrier, and the differences in intestinal immunity. Studies have shown that glycosyltransferase (*FUT8*) changes core fucosylation and catalyzes the transfer of α-1,6-bound fucose to the innermost N-acetylglucosamine layer in order to complete core fucosylation [5]; as such, the biological function of core fucosylation in mammalian cells has been extensively studied. *FUT8* is widely involved in a variety of life processes, including in the differential diagnosis of tumors [6], cancer [7], immune response [8,9], the regulation of gut microbiota [10], and cell adhesion [11]. In addition, it has been shown that down-regulation of the *FUT8* expression inhibits the invasion and migration ability of breast cancer cells [12]. Some studies have shown that key proteins regulated by *FUT8* play an important role in immune responses and cell adhesion [13]. Recently, it has been shown that by inducing trinitrophenol and lipopolysaccharide, the degree of inflammation in *FUT8*-deficient (FUT8 −/−) mice showed significantly lower inflammation in colitis than in wild-type (FUT8 +/+) mice [14,15]. Xiaoli et al. noted that insertion/deletion and duplication of SVS in genes such as *FUT8* and *MDM2* resulted in the loss of stop codon or frameshift mutation, as well as the aberrant alternative splicing of transcripts. These genes are involved in cell lamin filament, intermediate filament cytoskeletons, supramolecular complexes, cell differentiation, regulation of macromolecule metabolic processes, etc. [16]. However, the roles of *FUT8* regulating *E. coli* F18 susceptibility in IPEC-J2 cells remains unclear. 

The promoter region consists of transcription start sites (TSSs) that recognize and specifically bind cis-acting elements in the promoter region. Meanwhile, single-nucleotide polymorphisms (SNPs) and base mutations in the promoter region cause changes in genetic expression [17]. The concept of single-nucleotide polymorphisms, which has been seen as one of the hotspots in the field of life science, was put forward in the early 1990s. SNPs and single-nucleotide mutations are due to substitutions of only one base. SNPs or mutations can be associated with susceptibility to disease, pathogenesis, and the efficacy of specific drugs. Clinical detection of SNPs or mutations is important [18]. SNPs could be responsible for individual diversity, genome evolution, the most common familial traits, difference in drug responses between individuals, and complex and common diseases (such as diabetes, obesity, hypertension, and psychiatric disorders [19]). Nevertheless, the regulatory mechanism of the pig *FUT8* promoter in *E. coli* F18 is not fully understood. 

In this study, we systematically investigated the relationship between *FUT8* expression and *E. coli* resistance at the cellular and tissue levels. Then, we explored the effect of the *FUT8* gene on signaling pathways and tight junctions by using *FUT8* knockdown and transcriptome sequencing. Moreover, we obtained the sequences of the *FUT8* promoter region from the NCBI (http://www.NCBI.Nlm.nih.govn/, accessed on 7 August 2022) database and identified the core promoter of *FUT8* via luciferase assay. Additionally, we screened for SNP mutations in the promoter region of the *FUT8* gene in Meishan pigs and analyzed the effect of key transcription factors in promoter regions on SNP mutation sites. This study elucidated the mechanism of *FUT8* regulation on *E. coli* F18 resistance and provides a theoretical basis of strategies for the bioengineering regulation in *E. coli* F18 resistance of pigs.

## 2. Results

### 2.1. Association Analysis of FUT8 Expression and E. coli F18 Infection

**Figure 1:**
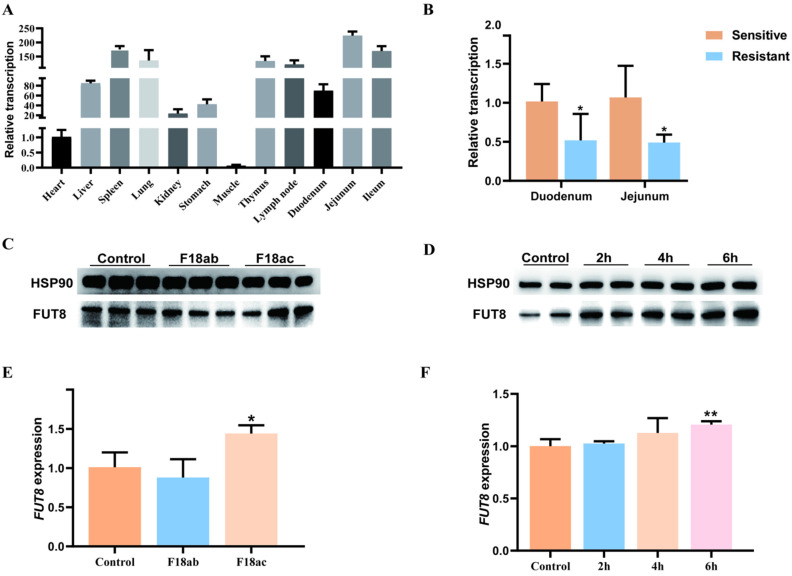
The relationship between the expression of *FUT8* and *E. coli* infection was analyzed at the tissue and cell levels. (**A**) Expression of the *FUT8* gene in 12 tissues of 35-day-old Meishan pigs. (**B**) Differential expression analysis of the *FUT8* gene in intestinal tissues between *E. coli* F18-resistant and -sensitive Meishan piglets. (**C**) Expression levels of *FUT8* in IPEC-J2 cells with *E. coli* infection were determined by Western blotting. (**D**) *FUT8* expression analysis in IPEC-J2 cells was determined using Western blotting, which was induced by 1 µg/mL LPS at 0 h, 2 h, 4 h, and 6 h. (**E**) Expression of *FUT8* in IPEC-J2 cells with *E. coli* infection was determined by RT-qPCR testing. (**F**) *FUT8* expression in IPEC-J2 cells with 1 µg/mL LPS at 0 h, 2 h, 4 h, and 6 h induction was determined using RT-qPCR testing. * Represents a significant difference (*p* < 0.05) and ** represents an extremely significant difference (*p* < 0.01).

In order to preliminarily investigate the role of *FUT8* in the resistance of piglets to *E. coli* F18 infection, we detected the expression level of *FUT8* in different tissues of 35-day-old Meishan pigs and the duodenal tissues of *E. coli* F18-sensitive and -resistant piglets. RT-qPCR detection showed (Figure 1A) that *FUT8* was expressed in the 12 tissues tested, with high expression in the spleen, lung, thymus, lymph node, jejunum, and ileum, but low expression in heart and muscle tissues (Figure 1A). In addition, the expression of *FUT8* in the duodenum and jejunum of the resistant group was significantly down-regulated (*p* < 0.05), as shown in Figure 1B. Moreover, immunohistochemical analysis showed that *FUT8* was mainly distributed in small intestinal epithelial mucosa cells of sensitive piglets (Figure S1). Then, we further analyzed the expression changes in *FUT8* within IPEC-J2 cells upon *E. coli* F18 invasion. Through the processes of Western blotting (Figure 1C) and RT-qPCR (Figure 1E) testing, it was observed that the expression of *FUT8* was significantly increased in F18ac-treated cells. To explore the role of *FUT8* in IPEC-J2 cells, we detected the subcellular localization of *FUT8* using nuclear-cytoplasmic fractionation. We observed that FUT8 was present primarily in the cytoplasm of normal IPEC-J2 cells or *E. coli* F18-infected IPEC-J2 cells (Figure S2). Within *Escherichia coli*, LPS was used to stabilize the outer membrane of this bacterium; further, LPS-induced IPEC-J2 cells were found to induce cellular immune responses. Our findings demonstrated that *FUT8* was significantly up-regulated in IPEC-J2 cells after LPS induction for 6 h (Figure 1D,F), which suggested that *FUT8* may play a critical role in regulating *E. coli* F18-induced immune responses.

### 2.2. Establishment of IPEC-J2 Cell Lines with FUT8 Interference

**Figure 2:**
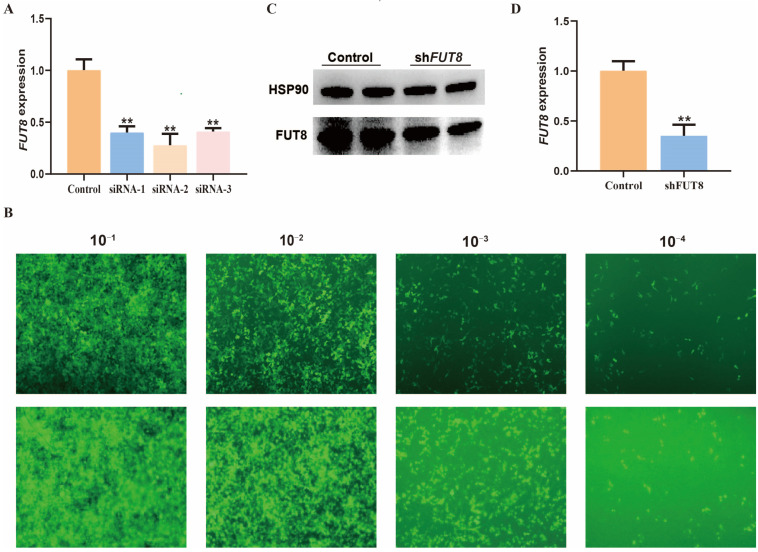
Establishment of cell lines with *FUT8* interference. (**A**) The interference efficiency of siRNA on the *FUT8* gene within IPEC-J2 cells. (**B**) Detection of lentiviral titer (24 h, 40×). (**C**,**D**) Knockdown efficiency of the *FUT8* gene in shFUT8 cells, which was determined using Western blotting (**C**) and RT-qPCR (**D**) tests. ** *p* < 0.01.

In order to understand the regulatory role of *FUT8* in IPEC-J2 cells, we constructed three siRNA vectors for the *FUT8* gene and determined that the optimal interference efficiency of *FUT8* in IPEC-J2 cells was 62.5% via RT-qPCR testing, as shown in Figure 2A (*p* < 0.01); furthermore, this interference sequence siRNA was selected for lentiviral packaging. The recombinant interfering lentiviral vector and packaging plasmid were co-transfected with 293T cells and, after 24 h of incubation, 293T cells expressed green fluorescence that indicated that the lentiviral packaging was successful. The virus concentrates were collected for titer determination, which is shown in Figure 2B. Further, the expression of green fluorescent was observed in 10^−1^, 10^−2^, 10^−3^, and 10^−4^ virus concentrates. Moreover, the number of fluorescent cells gradually decreased with increasing dilution. According to the fluorescent cells observed in the 10^−4^ virus concentrate, the virus titer was calculated to be 4 × 10^8^ TU/mL, which met the concentration requirements for cell infection. We found that the transcription and protein levels of *FUT8* were significantly downgraded in the shFUT8 group (*p* < 0.01), which indicated that the IPEC-J2 cell line with stable interference of *FUT8* was successfully constructed by the Western blotting (Figure 2C) and RT-qPCR (Figure 2D) tests.

### 2.3. Effect of FUT8 Knockdown on the Adhesion Ability of E. coli F18

**Figure 3:**
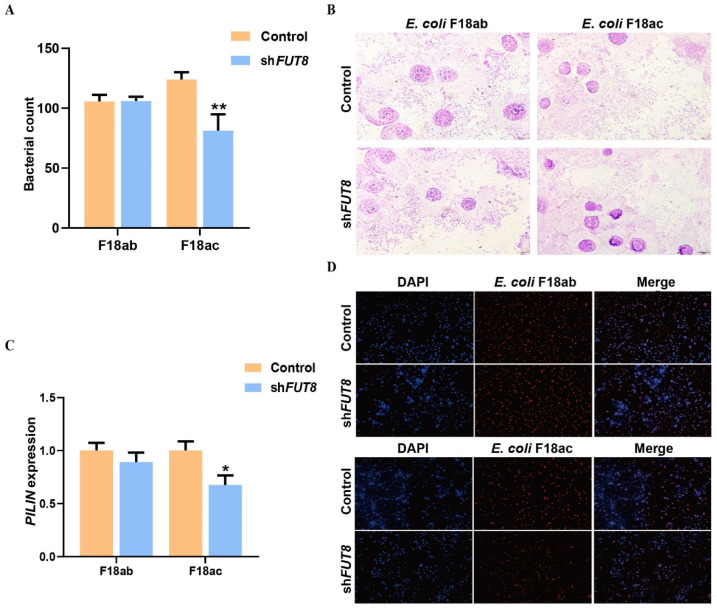
Analysis of the effect of *FUT8* expression on *E. coli* adhesion. (**A**) Expression detection of *E. coli* F18 fimbriae gene (*PILIN*) via relative quantification in *FUT8*-silenced IPEC-J2 cells. (**B**) Gram staining assay. An optical microscope (400×) was used to observe cells. (**C**) Colony count for F18ab and F18ac adhered shFUT8 cell. (**D**) Indirect immunofluorescence assay, blue fluorescence indicates nuclear staining via DAPI, and red fluorescence indicates staining with the anti-*E. coli* antibody. Cells were observed under a fluorescence microscope. * *p* < 0.05 and ** *p* < 0.01.

Regarding future insight into how *FUT8* regulates susceptibility to *E. coli* F18 infection, we evaluated the effect of *FUT8* expression on the level of adhesion of *E. coli* F18-expressing fimbriae to IPEC-J2 cells. Colony counting (Figure 3A), and expression detection of F18-fimbriae protein (PILIN) (Figure 3C), showed significantly lower numbers of *E. coli* F18ac adhering to IPEC-J2 cells in the shFUT8 group (*p* < 0.01). Further, there was no significance in the *E. coli* F18ab invasion (*p* > 0.05). In addition, the adhesion ability of *E. coli* F18ac adhesion to the IPEC-J2 cells was also assessed by Gram staining (Figure 3B), indirect immunofluorescence (Figure 3D), and scanning electron microscopy (Figure S3). Our results showed that *FUT8* knockdown significantly decreased the level of *E. coli* F18ac adhesion at IPEC-J2 cells.

### 2.4. Transcriptome Sequencing Analysis of IPEC-J2 Cells after FUT8 Knockdown by RNA-seq

**Figure 4:**
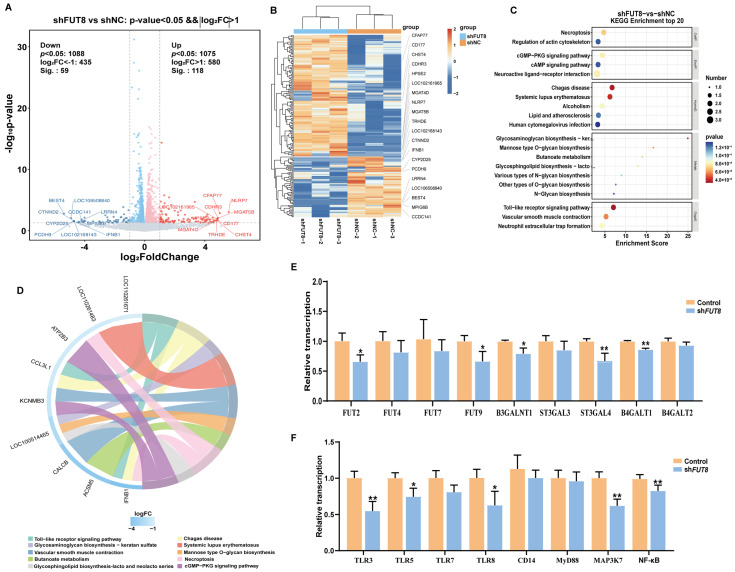
Identification and analysis of differentially expressed genes (DEGs) in IPEC-J2 cells following *FUT8* knockdown. (**A**) Statistical analysis of the number of up- and down-regulated DEGs identified between the shFUT8 and shNC samples. (**B**) Heat map analysis of DEGs. (**C**) KEGG pathway enrichment analysis for the DEGs. (**D**) Visualization analysis of key pathways and key DEGs. (**E**,**F**) Effect of *FUT8* knockdown on the expression level of glycosphingolipid biosynthesis and Toll-like signaling pathway genes was analyzed using RT-qPCR testing. * *p* < 0.05 and ** *p* < 0.01.

We next investigated the molecular mechanism of the *FUT8* gene in the regulation of *E. coli* F18 infection. We performed a comparative transcriptome sequencing of six RNA libraries (shFUT8-1, shFUT8-2, shFUT8-3, shNC-1, shNC-2, and shNC-3) using the Illumina HiSeq 2500 platform. A total of 299.65 and 300.26 million clean reads after filtering were obtained from the shFUT8 and shNC libraries, respectively (see Supplementary Table S1). A |log2 (fold change)| >1 and *p* < 0.05 were used as the standard thresholds for screening the differentially expressed genes (DEGs). A total of 177 DEGs were obtained, of which 118 were up-regulated (66.67%) and 59 were down-regulated (33.33%) in the shFUT8 group (see Supplementary Table S2; Figure 4A). The expression profiles of the DEGs in the two groups were visualized using a heat map; the DEGs in the shFUT8 and shNC samples were clustered separately, whereas for each of them the three replicates were clustered together (Figure 4B). Further gene ontology (GO) function annotations of transcripts were shown in Figure S4. Kyoto Encyclopedia of Genes and Genome (KEGG) enrichment analyses were performed in order to explore the potential functions of the DEGs (Figure 4C). Furthermore, these DEGs were enriched in 20 pathways, including 41 significantly enriched Toll-like receptor signaling pathways (i.e., *p* < 0.05) (see Supplementary Table S3). Among them, the pathway of the “Glycosphingolipid biosynthesis-lacto and neolacto series” (ko00940) was enriched in the shFUT8 and shNC samples (Figure 4D), which probably played an important role in the regulation of *E. coli* F18 receptor formation [20,21].

To confirm the effect of *FUT8* on the key pathways in the IPEC-J2 cells, the expression of genes in the cellular Toll-like receptor signaling and glycosphingolipid biosynthesis pathways in the IPEC-J2 cells were examined via RT-qPCR testing. Results showed that the expressions of *FUT2*, *FUT9, B3GALNT1*, *STAGAL4*, and *B4GALT1* from the glycosphingolipid biosynthesis pathway were all significantly decreased (*p* < 0.05) in IPEC-J2 cells following *FUT8* knockdown (shown in Figure 4E). Additionally, the expressions of *TLR3*, *TLR5*, *TLR8*, *MAP3K7*, and *NF-κB* from the Toll-like receptor signaling pathway were also significantly down-regulated in *FUT8-*knockdown IPEC-J2 cells (*p* < 0.05, Figure 4F). The above results indicated that *FUT8-*mediated Toll-like receptor signaling and glycosphingolipid biosynthesis pathways may act as critical roles in the process of IPEC-J2 cell responses to *E. coli* F18 infection.

### 2.5. Analysis of the Effect of FUT8 on Tight Junction Genes Expression

**Figure 5:**
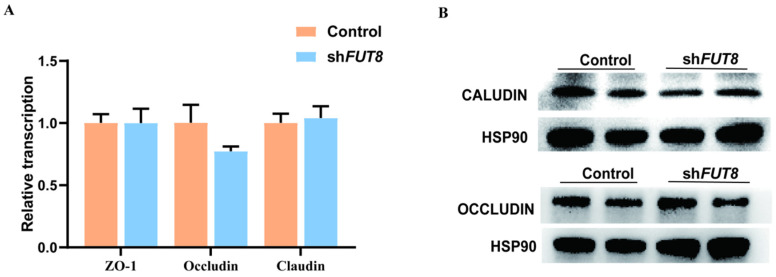
Effect of the *FUT8* genes on the expression levels of tight junction genes. (**A**) qRT-PCR analysis of mRNA expression of tight junction protein in *FUT8*-knockdown IPEC-J2 cells. (**B**) Western blot analysis of protein expression of tight junction genes in *FUT8*-knockdown IPEC-J2 cells. HSP90 was used as an internal reference.

In addition, we analyzed the expression changes in tight junction protein genes in *FUT8*-knockdown IPEC-J2 cells and found that there were no significant changes in the expression of mRNA (Figure 5A) and protein (Figure 5B) levels after *FUT8* knockdown (*p* > 0.05). These results indicated that *FUT8* knockdown may have no influence on the tight junction of IPEC-J2 cells.

### 2.6. Identification Analysis of Pig FUT8 Core Promoter Region

**Figure 6:**
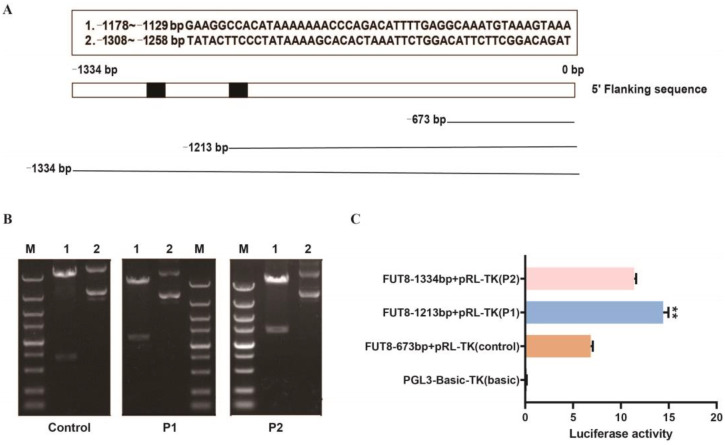
Impact of Pig *FUT8* on the core promoter region. (**A**) Prediction of *FUT8* core promoter region and truncation of detection fragment. (**B**) Agarose gel electrophoresis of PCR products that were digested by restriction enzymes. Lane 1: plasmid digested by *Kpn*I and *Xho*I; Lane 2: plasmid DNA; Lane M: DL5000 marker. (**C**) Luciferase assay of different vectors. Basic: negative control. ** *p* < 0.01.

To further explore the regulatory mechanism of *FUT8* expression, we focused on the promoter analysis of pig *FUT8* (GenBank: XM_005666322.3). The approximately 1300 bp sequence upstream of the transcription start site was used as a template in order to predict the core promoter region using BDGP software (https://www.fruitfly.org/seq_tools/promoter.html). We found two possible promoter regions (−1178~−1129 and −1308~−1258). According to the prediction results, the 1300 bp upstream sequence was divided into three fragments, namely, −673–0 bp (control), −1213–0 bp (P1), and −1334–0 bp (P2) (Figure 6A). As shown in Figure 6B, PCR amplification products were assessed by agarose gel electrophoresis. The luciferase assay showed that the luciferase intensity of pRL-P1 was significantly higher than that of the other transfected groups (Figure 6C). Our results suggested that the core promoter region of the pig the *FUT8* gene is located at −1213 bp to −673 bp.

### 2.7. Important SNP and Transcription Factor Identification Analysis of Pig the FUT8 Gene Promoters

**Figure 7:**
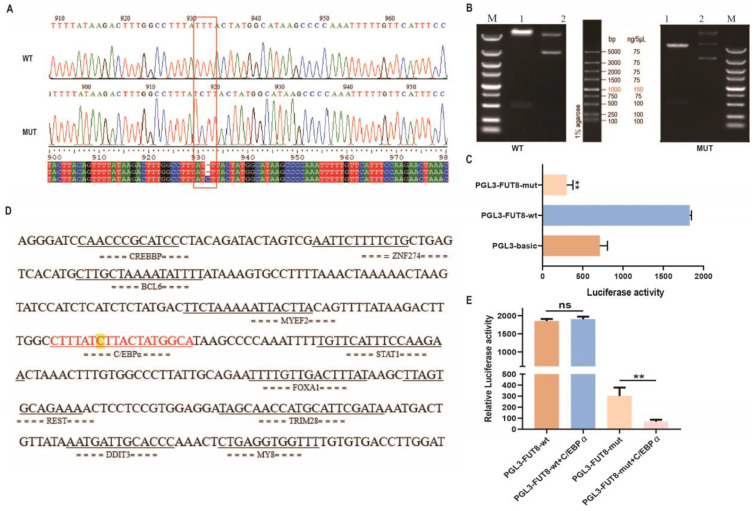
Promoter polymorphism and transcription factor analysis. (**A**) SNP mutation sites were identified via PCR sequencing, i.e., wild-type sequence and mutant sequences. Inserted sequences are marked with red boxes. (**B**) Mutant and wild-type sequences were verified by agarose gel. Lane 1: plasmid digested by *Kpn*I and *XhoI*; Lane 2: plasmid DNA, Lane M: DL5000 marker. (**C**) Relative luciferase activity of wild-type (wt) or mutant-type (mut) *FUT8* luciferase reporter. (**D**) Transcription factor binding sites were predicted from the insertional mutated sequences. (**E**) Effects of *C/EBPα* on *FUT8* transcriptional activity by dual-luciferase activity assay. ^ns^ *p* > 0.05 and ** *p* < 0.01.

In order to investigate the genetic variation in the promoter region of the pig *FUT8* gene, we sequenced the PCR product of this core promoter region in the DNA bulk of 400 Meishan pigs and detected a 1 bp (C base) insertion mutation at the −774 bp upstream of the *FUT8* gene, which was verified by sequencing; furthermore, the agarose indicated that the *FUT8* wild-type and mutant vectors were constructed successfully (Figure 7A,B). Next, we constructed wild-type and mutant vectors of *FUT8* and further transfected the recombinant plasmids of PGL3-FUT8-mut or PGL3-FUT8-wt sequences into 293T cells for luciferase activity analysis, as shown in Figure 7C. Results showed that the luciferase activity was significantly decreased in cells transfected with PGL3-FUT8-mut plasmids compared to the controls (*p* < 0.01). In order to further identify the important transcription factors in regulating *FUT8* expression, we presented the potential transcription factor binding sites such as *MY8*, *BCL6*, *STAT1*, REST, *C/EBPα*, and *CREBBP*, as shown in Figure 7D, of which *C/EBPα* was found in the 1 bp (C base) insertion mutation region of the pig *FUT8* gene. Herein, we performed dual-luciferase activity assays in order to investigate the effects of *C/EBPα* on the transcriptional activity within the *FUT8* promoter. As shown in Figure 7E, *C/EBPα* led to the inhibition of transcriptional activity. Our results indicated that the 1 bp C base insertion mutation at the −774 bp region probably inhibited the transcriptional binding activity of *C/EBPα* to the *FUT8* promoter, and then decreased the *FUT8* expression.

## 3. Discussion

**Figure 8:**
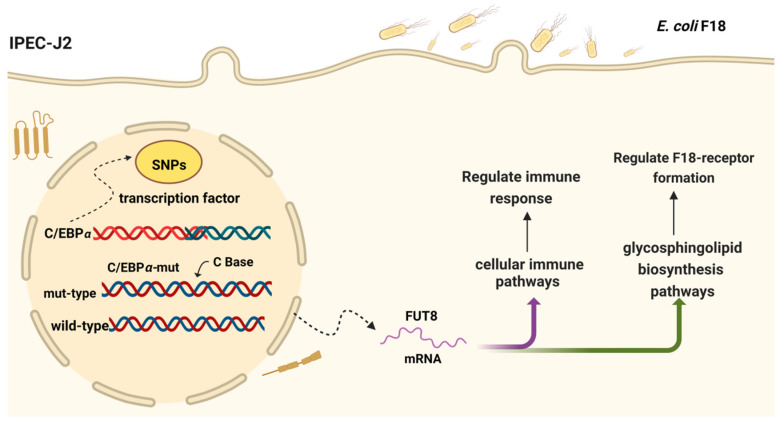
The diagram of the regulation mechanism of *E. coli* F18 infection of the *FUT8* gene in IPEC-J2 cells.

The major causative agent of post-weaning diarrhea (PWD) in piglets is enterotoxin-producing *Escherichia coli*, which is a Gram-negative bacterium. *E. coli* F18 binds to the cellular *E. coli* F18 receptor through bacterial fimbriae, which adheres to the IPEC-J2 and then produces LPS and enterotoxins, which damage the cells and impede the barrier function of the intestinal epithelial cells, leading to metabolic dysregulation and inflammatory reactions [22,23,24,25]. Therefore, the resistance of piglets to *E. coli* F18 exposure depends on the expression of the *E. coli* F18 receptor in the porcine intestinal epithelium and the ability of the body to regulate intestinal immunity. The potential regulatory role of *FUT8* in IPEC-J2 cells after *E. coli* F18 exposure was better understood by using IPEC-J2 to mimic the adhesion phenomenon of *E. coli* F18 in vitro. In this study, we successfully utilized IPEC-J2 cells in order to simulate the phenomenon of *E. coli* F18 adhesion in vitro. Moreover, it is crucial to enhance our comprehension of the underlying mechanisms behind *FUT8* regulating *E. coli* F18 susceptibility. Briefly, an SNP (C base insertion mutation) was detected in the −774 bp region of the *FUT8* core promoter (−1213 bp to −673 bp), and most likely inhibited the transcriptional binding activity of *C/EBPα* to the *FUT8* promoter, which then lead to the decrease in *FUT8* expression and the enhancing of the resistance to *E. coli* F18 infection (Figure 8).

Here, we identify an important regulatory role for *FUT8* in the regulating of susceptibility to *E. coli* F18, as well as glycosylation, which is an important mode of the post-translational modification of proteins. *FUT8* is a very important glycosyltransferase that catalyzes the modification of core protein fucose and plays an important role in regulating the normal physiological function of glycoproteins [26]. FUT8-catalyzed core fucosylation plays a role in a variety of life processes in the body and is involved in the regulation of a variety of physiological and pathological processes. Studies have shown that the core fucose level is increased in tumor tissues compared with normal tissues [27] and that the aberrant expression of *FUT8* promotes the proliferation and invasion of malignant tumors such as liver cancer, breast cancer, and non-small cell lung cancer [28,29,30]. It is also important in lectin-mediated cytokine production that is induced by immune cells, where the affinity of the antibody to the receptor is reduced by 98–99% after the Fc region of the antibody has been glycosylated by core rockweed [31]. Li et al. found that *FUT8* affected the intestinal microbiota profile of mice, with FUT8 −/− mice having a significantly different intestinal microbiota when compared to FUT8 +/+ mice, and the different microbiota were mainly Gram-negative bacteria [10]. Shinzaki et al. found that defects in *FUT8* resulted in a lack of core rockweed glycosylation of the TCR (T cell receptor) in the mouse intestinal mucosa, such that TCR-mediated inflammatory signals cannot be activated, thereby resulting in improved colonic inflammation in mice [32,33]. Currently, the research on *FUT8* has focused on tumor and intestinal inflammation—mainly in humans and mice—and there are few studies on the relationship between the expression level of the pig *FUT8* gene and the drug resistance of *Escherichia coli*.

Intestinal epithelial cells are the primary site of defense against invading heterologous pathogens that can directly react with Gram-negative bacteria and LPS; further, it is this process that results in the development of diarrhea in piglets [34,35]. Therefore, this study selected IPEC-J2 as the research object at the cellular level. First, we examined the expression of *FUT8* at the mRNA and protein levels following *E. coli* F18 infection and LPS induction by 1 µg/mL. The results showed that compared with the control group, the expression level of *FUT8* in IPEC-J2 cells infected with *E. coli* F18ac was significantly increased (*p* < 0.05), and the expression level of the *FUT8* gene had no significant change after infection with *E. coli* F18ab (*p* > 0.05). The expression of the *FUT8* gene increased in a stepwise manner after LPS induction by 1 µg/mL, and the expression of the *FUT8* gene was significantly increased after 6 h of LPS induction by 1 µg/mL (*p* < 0.01). LPS is an endotoxin released by *Escherichia coli* adhesion to intestinal epithelial cells and plays an important role in the inflammatory response. This suggests that the expression of the *FUT8* gene is closely related to the collective resistance of *E. coli* infection, and *FUT8* may be involved in the life process of *E. coli* F18 resistance regulation. Studies have shown that changes in intestinal mucosal glycosylation in patients with IBD affect the integrity of the intestinal mucosa, which is inseparable from bacterial damage to mucosal epithelial cells causing intestinal inflammation. In IBD patients, *FUT8* expression is higher in moderately to severely inflammatory mucosa than in mildly inflammatory mucosa [14,36]. These findings suggest that the high expression of *FUT8* may exacerbate bacterial exposure of intestinal cells, leading to an inflammatory response in the body.

In this study, in order to investigate the relationship between the expression of the *FUT8* gene and *E. coli* F18 resistance, the stable interference of the *FUT8* gene in IPEC-J2 cells was constructed; further, the effect of down-regulation of the *FUT8* gene expression on the ability of *E. coli* F18 to adhere to IPEC-J2 cells was comprehensively analyzed by the processes of bacterial counting, F18-fimbriae protein (*PILIN*), indirect immunofluorescence, Gram staining, and scanning electron microscopy. After *FUT8* gene interference, IPEC-J2 cells were infected with *E. coli* F18ac and *E. coli* F18ab; colony counting results showed that there was no significant change in the number of *E. coli* F18ab adhesion after *FUT8* gene interference; additionally, *E. coli* F18ac was significantly decreased (*p* < 0.01). F18-fimbriae protein (*PILIN*) showed a consistent trend with colony counts. Based on the above experimental results, we analyzed the adhesion ability of *E. coli* using indirect immunofluorescence techniques and Gram staining observation after the *E. coli* infection. The results showed that there was no significant difference in the *E. coli* F18ab group after *E. coli* infection; in addition, *E. coli* F18ac was significantly decreased. Fucosyltransferases in mammals can be divided into two categories according to the way they are linked to sugar chains, one is *FUT1* to *FUT11* transferases other than *FUT8*, and the other is *FUT8* unique fucosyltransferases [37,38]. Kashiwazaki et al. showed that *FUT9* knockout mice had very low virus titers after infection with the virus and did not change significantly after inoculation with endotoxin [39]. Cai et al. have experimentally demonstrated that low expression of the *FUT8* gene reduces cellular activities, such as adhesion binding and migration infection, and improves resistance to disease [40,41]. *FUT8* expression is increased in ovarian cancer and the down-regulation of *FUT8* significantly inhibits the invasion and spread of tumor cells [42,43]. Munkley et al. showed that core fucosylation increased locally at sites of intestinal inflammation, and that expression of *FUT8* was positively correlated with inflammation [44]. These studies have shown that fucosyltransferase is involved in a variety of life processes in the body. Moreover, *FUT8*—as the only core fucosyltransferase—plays an important role in various physiological activities such as cancer, cell proliferation, migration, and immune and inflammatory responses in the body. Therefore, we speculate that the down-regulation of *FUT8* expression may help pigs to resist *E. coli* infection, pathogen adhesion, and pathogen colonization through a series of signaling and immune regulations.

In order to verify that the *FUT8* signaling pathway plays a role in *E. coli* infection with IPEC-J2 cells, we used RT-qPCR to detect the expression levels of key genes in the signaling pathway. We found that the significantly down-regulated expressions of *TLR3*, *TLR5*, *TLR8*, *MAP3K7*, and *NF-κB* were all present in FUT8-knockdown IPEC-J2 cells. *MAP3K7* knockout mice have reduced production of inflammatory cytokines in cells [45]. Moreover, *TLR5* is a cell surface receptor for innate immunity after pathogens invade the body. It is a receptor for the recognition of Gram-negative bacterial flagellin by the organism and plays an important regulatory role in *E. coli* invasion into the organism [46]. Low expression of *TLR5* helps to inhibit immune responses, reduce cell damage, and promote resistance to *Escherichia coli* infection in weaned piglets [47]. *TLR8* recognizes the nucleic acid components of microorganisms, which in turn initiate antiviral immune responses and promote the expression of inflammatory cytokines [48]. Additionally, we found that *FUT8* also significantly affected the glycosphingolipid biosynthesis pathways. Studies showed that glycosphingolipid biosynthesis correlated with the generation of the receptor for *E. coli* F18 [19,20]. Thus, we speculated that *FUT8* most likely regulated *E. coli* F18 susceptibility via the activation or suppression of TLR signaling (which is related to immune response) and glycosphingolipid biosynthesis (which is related to the formation of the *E. coli* F18 receptor). 

In this study, the core promoter region was predicted and the presence of the core promoter region was verified using a dual-luciferase assay. In order to investigate the genetic variation in the promoter sequence of the pig *FUT8* gene, we sequenced the PCR product of the *FUT8* gene promoter sequence and detected a 1-bp insertion mutation at the −774 bp upstream of the *FUT8* gene in Meishan pigs. Then, we constructed wild-type and mutant vectors with this base insertion. Through conducting this and performing dual-luciferase assays, it was revealed that the 1-bp insertion inhibited transcriptional activity. In order to investigate whether the 1-bp insertion affects the binding of the *FUT8* promoter DNA sequence to the transcription factor, we predicted the transcription factors that may bind to the insertion sequence and performed a dual-luciferase assay, which showed that co-transfection with *C/EBPα* resulted in significantly lower activity in the mutant than in the wild-type. The transcription factor CCAAT enhancer binding protein alpha (*C/EBPa*) is critical for cell proliferation; in addition, evidence suggests that *C/EBPα* may negatively control cell proliferation. *C/EBPa* is essential for granulocyte formation and is regulated by multiple mechanisms in acute myeloid leukemia, which is one of the major regulators of granulopoiesis [49]. During granulocyte formation, *C/EBPα* regulates differentiation at several steps, including the transition from common myeloid progenitor cells to granulocyte-macrophage progenitor cells [50]. Ayumi Hashimoto et al. demonstrated that therapeutic up-regulation of the transcription factor *C/EBPα* causes the inactivation of immune-suppressive myeloid cells inactivating and is an effective antitumor response across different tumor models and cancer patients [51]. This author’s finding, i.e., that the DNA-binding variant of the wild-type *C/EBPα* inhibited the formation of 3T3-L1 preadipocyte colonies, prompted further experiments with estradiol-regulated C/EBPα-ER, which was found to directly inhibit 3T3-L1 cell cycle progression [52]. More and more studies indicate that *C/EBPα* is down-regulated by mechanisms in acute myeloid leukemia, thereby highlighting that *C/EBPα* is a myeloid tumor suppressor [53]. Combined with the analysis of the above experimental results, we preliminarily proposed that the low expression of *FUT8* would inhibit *E. coli* F18 adhesion cells and ultimately improve the resistance of piglets to *E. coli*. However, further electrophoretic mobility shift assays (EMSA) and co-immunoprecipitation (CoIP) assays should be performed to determine the binding of C/EBPα to the *FUT8* promoter. Moreover, we will explore in depth the function of *FUT8* by RNA overexpression and CRISPR/Cas9 knockout. Our study will help to solve the problem of breeding screening for *E. coli* resistance in local pig breeds in China and provides a theoretical basis for the genetic breeding of *E. coli* resistance in the future.

## 4. Materials and Methods

### 4.1. Ethics Statement

All the experiments were approved by the Institutional Animal Care and Use Committee (IACUC) of Yangzhou University (pig: SYXK (Su) 2012-0029) and were performed according to the Animal Ethics Procedures and Guidelines of the People’s Republic of China. 

### 4.2. Reagents and Animal Material

The following antibodies were used for indirect immunofluorescence assay (IFA) and Western blot analysis: *E. coli* (ab137967, rabbit, 1:200) and HSP90 (ab59459, mouse, 1:500), which were both purchased from Abcam (Shanghai, China). FUT8 (NBP1-79869, rabbit, 1:1000) was purchased from NOVUS Ltd. Co. (Littleton, CO, USA). Additionally, IgG (Q6005, rabbit, 1:200) was from Dia-an biotech (Wuhan, China). Experimental pigs (Meishan) were acquired from Kunshan Conservation Ltd(Jiangsu, China). In the previous study, the experimental piglets were challenged with a daily dose of 4.6 × 10^8^ CFU of *E. coli* F18 strain, and the differences in susceptibility were assessed by assays, such as *E. coli* F18 bacteria counting and histopathological and in vitro adherence assays of intestinal porcine epithelial cells [54]. Ultimately, we selected three resistant piglets and three sensitive piglets for further analysis. All the experimental pigs were euthanized via the intravenous injection of pentobarbital sodium and duodenum, liver, heart, spleen, lung, stomach, kidney, muscle, lymph node, and thymus. Furthermore, the jejunum tissues of the 35-day-old Meishan pigs were collected, followed by storing them in liquid nitrogen in situ for further use.

### 4.3. E. coli F18 Exposure and LPS Induction

IPEC-J2 and *E. coli* F18ab and F18ac organisms were maintained in our laboratory and LPS was purchased from Sigma, USA. In this study, *E. coli* F18 and LPS-induced cells were used to detect the expression of *FUT8* at the mRNA and protein levels, respectively. IPEC-J2 cells were seeded in 12-well plates at a density of 5 × 10^4^ cells per well and cell culture medium was used to dilute E. coli to 1.0 × 10^9^ CFU/mL and then stimulated for 4 h, which is when the cell density reached about 80%. LPS by 1 µg/mL was induced at four time points: 0 h, 2 h, 4 h, and 6 h, and the group was set with three replicates in each group.

### 4.4. Primer Design and Sequence Synthesis

Quantitative PCR primers were based on their coding sequences in the NCBI (http://www.NCBI.Nlm.nih.govn/ (accessed on 1 May 2022)) database. The *GAPDH* gene was used as an internal reference gene. All the primers are shown in Supplementary Table S4.

### 4.5. Cell Culture 

IPEC-J2 was seeded into a 12-well plate at a density of 5 × 10^4^ per well; three designed pairs of siRNA sequences (see Supplementary Table S5) were transfected into cells when the density reached approximately 60%. Moreover, the expression of *FUT8* was detected by RT-qPCR testing. Gateway recombination technology was used in order to screen the siRNA-2 and the interfering vector was cloned into the lentiviral vector. After 24 h, the expression of green fluorescent was observed and puromycin was added to screen the positive cells. When the positive cells were stably expressed, the cells were collected for mRNA and protein detection in order to analyze the expression of the *FUT8* gene in IPEC-J2 cells.

### 4.6. RT-qPCR Analysis 

Total RNA was extracted from IPEC-J2 cells and tissues using a Trizol reagent (Takara, Dalian, China). The purity, as well as the total RNA concentration, was evaluated via a NanoDrop 1000 (Thermo, Massachusetts, USA); then, the RNA was kept at −80 °C. The synthesis of cDNA was performed via 4 µL of 5 × HiScript III qPCR SuperMix II (Vazyme Biotech Co., Ltd., Nanjing, China) along with a total RNA of 1000 ng and RNase-free ddH2O. RT-qPCR was performed in a 20 µL reaction system consisting of 2 µL Cdna (100–500 ng), 0.4 µL upstream and downstream primers (10 µmol/L), 10 µL Hieff UNICON^®^ Universal Blue qPCR SYBR Green Master Mix (Yeasen, Shanghai, China), and 7.2 µL double-distilled water. All experiments were performed in triplicates. The RT-qPCR instrument ABI7500 was employed for the evaluation of qPCR and the parameters were as follows: 95 °C for 5 min, 40 cycles of 95 °C for 10 s, and 60 °C for 30 s. Relative expression was calculated by the 2^−∆∆Ct^ method and interference efficiency was calculated by 1–2^−∆∆Ct^.

### 4.7. Western Blot Analysis

Total proteins were extracted using RIPA lysate (Cwbio, Beijing, China) from Thermo Fisher Scientific. A bicinchoninic acid (BCA) kit from Nanjing Keygen Biotech (Nanjing, China) was used for normalizing the protein levels (Biosharp, Beijing, China). The SDS-PAGE of the protein samples was subjected to electrophoresis at 120 V for 90 min in 10% gel; then, the protein sample was transferred to a PVDF membrane. Subsequently, they were blocked with skimmed milk powder and incubated with the corresponding primary antibody (1:1000) overnight at 4 °C. This was then followed by incubation of the corresponding secondary antibody (goat anti-mouse IgG HRP, goat anti-rabbit IgG HRP; CWBIO, Beijing, China). HSP90 (1:5000, Proteintech Group, Inc, Rosemont, IL, USA) was used as an equivalent protein internal reference.

### 4.8. Subcellular Fraction Extraction

Nucleocytoplasmic RNA of IPEC-J2 was isolated and extracted using the Cytoplasmic and Nuclear RNA Purification Kit (Norgen Biotek, Thorold, ON, Canada).

### 4.9. Immunohistochemical Analysis

Jejunum tissues were collected from *E. coli* F18-resistant and -susceptible individuals. Serial sections of paraffin-embedded tissues were first treated with 3% peroxide for 15 min to quench endogenous peroxidase. The sections were rinsed for 15 min with 0.01M PBS (Jiangsu KeyGEN BioTECH Corp., Ltd.) and then incubated at room temperature for 2 h with the following primary antibodies: rabbit anti-FUT8. Whereafter, they were sequentially incubated with the HRP conjugated secondary antibody (Jackson ImmunoResearch Laboratories, West Grove, PA, USA., 1:50) for 30 min at room temperature with diaminobenzidine (DAB) as the substrate chromogen (Jiangsu KeyGEN BioTECH Corp, Jiangsu, China), the sections were counterstained with hematoxylin.

### 4.10. In vitro Adherence Assays with E. coli F18 

IPEC-J2 cells were inoculated into 12-well plates at a density of 5 × 10^4^ per well and placed in a constant temperature incubator at 37 °C. *E. coli* F18ab and *E. coli* F18ac fimbriae standard strains were inoculated into the LB culture medium and then incubated for 12 h on a rotating shaker table (200 rpm). PBS was used for the wash and the bacteria collected was centrifuged at a rotating speed of 4000 rev/min for 10 min at three times; next, the cell culture medium was used to dilute *E. coli* to 1.0 × 10^9^ CFU/mL, and then added to 12-well culture plates. Cells were cultured for 4 h in an incubator under the conditions of 37 °C; further, F18-fimbriae protein, colony count, Gram staining, scanning electron microscopy (SEM), and indirect immunofluorescence assays were all used in order to detect *E. coli* adherence to cells [47,55,56].

### 4.11. Indirect Immunofluorescence (IFA)

IPEC-J2 cells (*E. coli* F18ab exposure, *E. coli* F18ac exposure, shFUT8, and control) were gently washed three times with PBS and were then fixed with 4% paraformaldehyde (Solarbio, Beijing, China) for 0.5 h. Next, they were washed with PBS three times again, then the cells were treated with 500 μL 0.5% Triton X-100 for 15 min. Then, washing with PBS and goat blocking serum was added, they were incubated for 2 h, and then the cells were incubated with an *E. coli* antibody (1:200) (Gene Tex, San Antonio, TX, USA) and secondary antibody IgG (1:200) (Affinity, Cincinnati, OH, USA), respectively. Subsequently, the cells were incubated with DAPI (1:1000) (Solarbio, Beijing, China), and then washed three times with 0.5% PBST. Finally, cells were mounted using a fade-resistant fluorescent medium and were analyzed immediately by confocal microscopy.

### 4.12. Single-Nucleotide Polymorphisms in the Porcine FUT8 Gene Promote 

According to the promoter sequence of the porcine *FUT8* gene (GenBank: XM005666322.3), the PCR primers were designed by Prime Premier 5.0 software. PCR assays were conducted in a 50 µL volume containing 200 ng of the DNA template, 10 µL of PCR Master Mix, and 25 pmol of each forward and reverse primer. The thermal conditions were as follows: 95 °C for 5 min, 36 cycles of 95 °C for 30 s, 55 °C for 30 s, 72 °C for 60 s, and a final extension at 72 °C for 10 min. The PCR products were checked by electrophoresis in a 2% agarose gel, and then sequenced by Sangon Biotech (Sangon, Shanghai, China).

### 4.13. Dual-luciferase Reporter Assays

The 2000-bp upstream sequence of the transcription start site of the pig *FUT8* gene (GenBank: XM005666322.3) was obtained from the NCBI (http://www.NCBI.Nlm.nih.govn/, accessed on 7 August 2022) database and the BDGP (http://www.fruitfly.org/seq_tools/promoter.html, accessed on 7 August 2022) was used in order to predict the core promoter region. Different promoter fragments were ligated into the pGL3-Basic vector in order to construct recombinant plasmids. Positive clones of recombinant plasmids were examined by sequencing (Shanghai Bioengineering Co., Ltd., Shanghai, China). When the density of 293T cells reached a 70–80% confluence in the 12-well plates, the expression vector and pRL-TK vector were co-transfected into the cells. Dual-luciferase assay was performed in strict accordance with the kit instructions. Luciferase activity was analyzed on a luminometer equipped with a Dual-Luciferase Assay System (Promega Corporation, Madison, WI, USA). The relative fluorescence intensity was calculated as Firefly Luc (Ff)/Renilla-Luc (Rn). The transcription factors in the core promoter region of *FUT8* were predicted using the Alibaba software.

### 4.14. Transcription Factor Prediction

Alibaba (http://www.gene-regulation.com/pub/programs/alibaba2/index.html?, accessed on 16 August 2022) and TFDB (http://bioinfo.life.hust.edu.cn/AnimalTFDB/, accessed on 16 August 2022) were used in order to screen out the important transcription factor binding sites (*C/EBPα*), which were based on the sequence of the *FUT8* promoter region, as well as in the conducted validation experiments by dual-luciferase activity assay.

### 4.15. Statistical Analysis

In order to conduct statistical analyses, the SPSS v.20 software (IBM Corp, Armonk, NY, USA) and GraphPad Prism 6.0 software (GraphPad Inc., La Jolla, CA, USA) were used. The relative quantitative results were examined via the 2^−∆∆Ct^ method. The analysis was performed using Student’s *t*-test (two-tailed). * *p* < 0.05 and ** *p* < 0.01 were considered statistically significant.

## 5. Conclusions

In conclusion, our study showed that the down-regulation of *FUT8* correlates with *E. coli* F18 resistance. Moreover, we found that the insertion mutation (1 bp C base) located at −774 bp in the *FUT8* promoter (−1213 bp to −673 bp) probably inhibited *FUT8* expression by affecting the binding of C/EBPα to the *FUT8* promoter, thereby improving the *E. coli* F18 resistance in piglets. Our results revealed the regulatory mechanism of the pig *FUT8* gene affecting the resistance to *E. coli* F18 infection. This finding will provide a theoretical reference for the molecular breeding of combating bacterial diarrhea in Chinese local pig breeds.

## Data Availability

The data presented in this study are available on request from the corresponding author.

## Supplementary Materials

The following supporting information can be downloaded at: https://www.mdpi.com/article/10.3390/ijms232314713/s1.

## Abbreviations

*FUT8*	Fucosyltransferase 8
PWD	Post-weaning diarrhea
*E. coli*	*Escherichia coli*
IPEC-J2	Intestinal porcine epithelial cell
IFA	Indirect immunofluorescence assay
SEM	Scanning electron microscopy
LPS	Lipopolysaccharide
FBS	Fetal bovine serum
DMEM	Dulbecco’s modified Eagle’s medium
NF-κB	Nuclear factor κB
SNPs	Single-nucleotide polymorphisms
DEGs	Differentially expressed genes
TSSs	Transcription start sites
KEGG	Kyoto Encyclopedia of Genes and Genome
EMSA	Electrophoretic mobility shift assay
CoIP	Co-immunoprecipitation
TU	Transducing units
