# Supplementary material for: Effect and Mechanism Analysis of Pig FUT8 Gene on Resistance to Escherichia coli F18 Infection
Source: Int J Mol Sci. 2022 Nov 25;23(23):14713. doi: 10.3390/ijms232314713 (PMC9739813; doi:10.3390/ijms232314713)
Supplement: Supplementary file 1 [file ijms-23-14713-s001.zip › ijms-1980572-supplementary Figure S1-S4.pdf]

Supplementary Figures

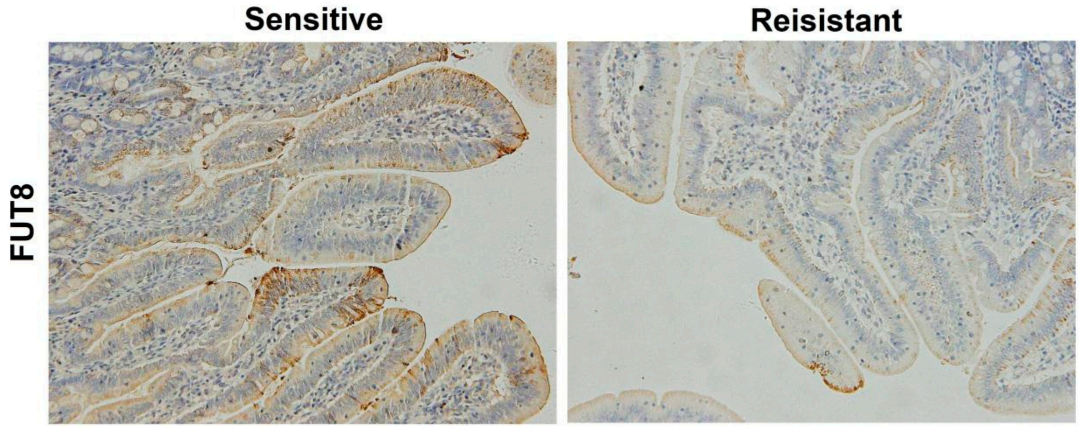

Figure S1. Immunohistochemical analyses of FUT8 in jejunum tissue between F18-resistant and -sensitive piglets. (100×, scale bar = 10 μm).

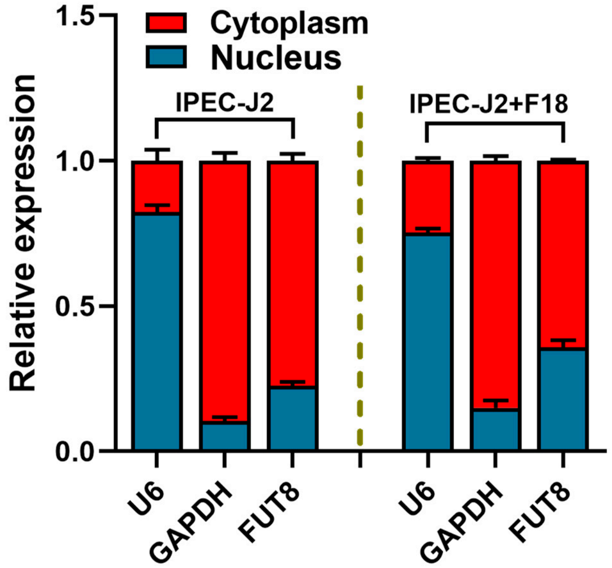

Figure S2. Nuclear-cytoplasmic fractionation assay in normal IPEC-J2 cells and *E. coli* F18-infected IPEC-J2 cells. *GAPDH* was considered as a cytoplasmic protein control and *U6* was used as a nuclear control.

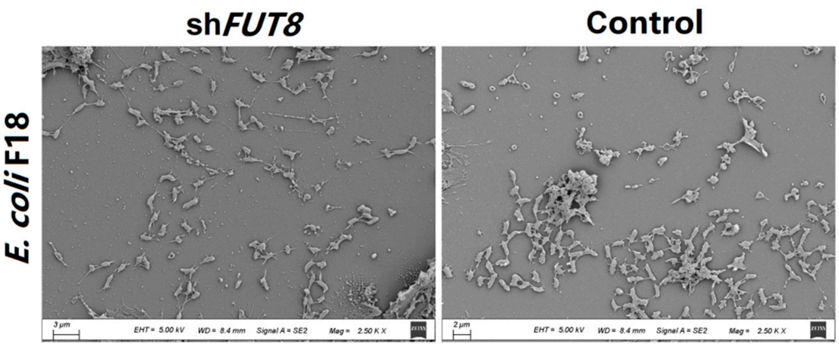

Figure S3. Scanning electron microscopy (SEM) assay, IPEC-J2 cells were observed under a scanning electron microscope (2000×).

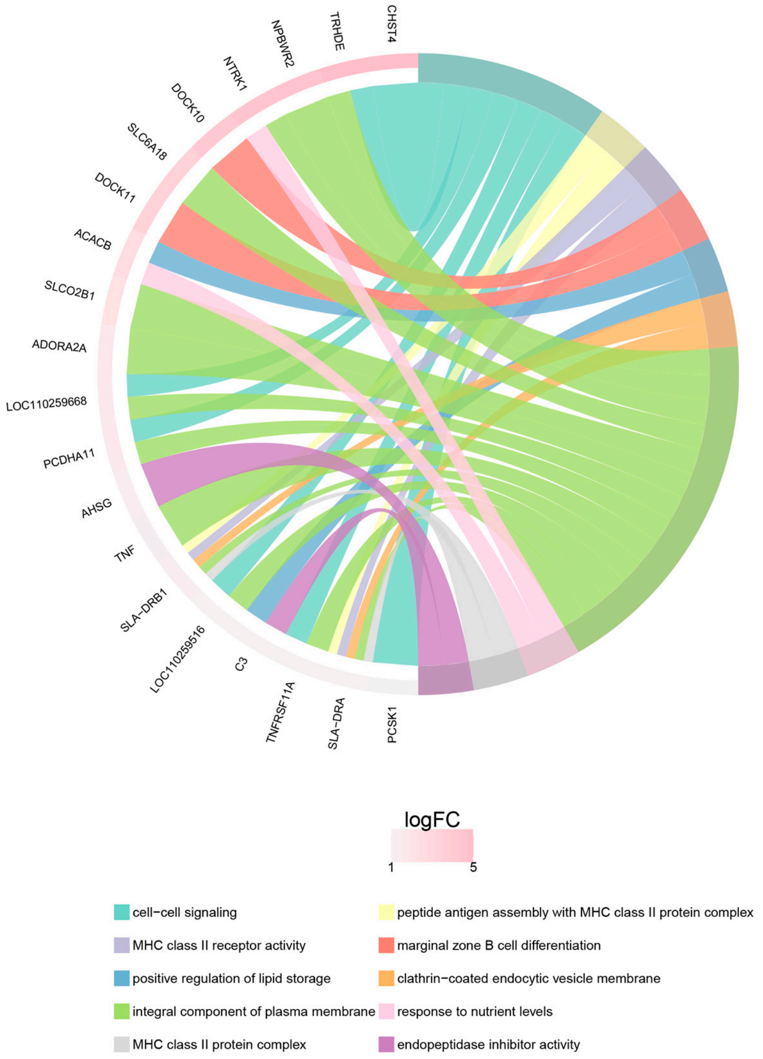

Figure S4. Visualization analysis of Gene ontology (GO) terms and key differentially expressed genes (DEGs).
